# Terahertz Multiple Echoes Correction and Non-Destructive Testing Based on Improved Wavelet Multi-Scale Analysis

**DOI:** 10.3390/s22093477

**Published:** 2022-05-03

**Authors:** Weihua Xiong, Lijuan Li, Jiaojiao Ren, Jian Gu, Dandan Zhang, Junwen Xue

**Affiliations:** 1 School of Optoelectronics Engineering, Changchun University of Science and Technology, Changchun 130000, China; w59u1f@163.com (W.X.); zimengrenjiao@163.com (J.R.); jokin_gezi@163.com (J.G.); zhangdandan_rita@126.com (D.Z.); xuejunwen001@126.com (J.X.); 2 Key Laboratory of Optoelectronic Measurement and Control and Optical Information Transmission Technology, Ministry of Education, School of Optoelectronic Engineering, Changchun University of Science and Technology, Changchun 130022, China; 3 Zhongshan Research Institute, Changchun University of Science and Technology, Zhongshan 528403, China

**Keywords:** time-of-flight, multiple echoes, multi-scale analysis, non-destructive testing

## Abstract

During terahertz (THz) non-destructive testing (NDT), multiple echoes from the sample interface reflection signals are mixed with the detection signals, resulting in signal distortion and affecting the accuracy of the THz NDT results. Combined with the frequency property of multiple echoes, an improved wavelet multi-scale analysis is put forth in this paper to correct multiple echoes, allowing the maximum retention of detailed signal information in contrast with the existing echo correction methods. The results showed that the improved wavelet multi-scale analysis enhanced the continuity and smoothness of the image at least twice in testing adhesive layer thickness, prevented missing judgments and misjudgments in identifying characteristic defects, and ensured accurate detection results. Hence, it is of great significance for evaluating the THz NDT results.

## 1. Introduction

Multiple echoes are common in terahertz (THz) non-destructive testing (NDT) [1], which results from uneven sample thickness [2], unreasonable system structure, inappropriate processing methods, etc. In recent years, the bonding quality inspection of the adhesive structure has become more crucial, as the adhesive structure of non-polar insulation sparse materials is widely used in the aerospace field [3]. THz is mostly the only means to inspect the bonding quality of non-polar insulation sparse materials, with its unique penetrating property. THz waves cannot penetrate the bonding samples in the aerospace field, since most of their substrates are made of metals or carbon fibers. Therefore, the detection information of bonding samples can only be obtained by reflective THz NDT [4]. The reflective terahertz nondestructive testing method is divided into inclination and collinear. When the sample thickness is large, the inclination terahertz nondestructive testing method cannot obtain the bonding information of the bonded layer [5]. Therefore, collinear terahertz time-domain spectroscopy is usually used in the detection of bonded samples in the aeronautical field [6]. When nondestructive testing of heat-resistant structure-bonded samples, it is unavoidable to introduce the multiple echoes caused by the surface echoes of the test samples reflected between the semi-reflective semi-lens and the focus lens, which are mixed in the detection signal, causing signal distortion and getting the wrong result of bonding quality of the bonded structure. Affects the accuracy of the Terahertz test results for bonded samples.

Researchers have been working on correcting multiple echoes in detecting THz time-domain spectroscopy systems. Hirsch et al. used the pulse transmission model in the transmitter of a THz time-domain spectroscopy system to inhibit the effects of multiple echoes generated from the testing samples [7]. This method corrects the multiple echo effects while reducing the details of the detected signals and the amount of information initially carried by those signals. Naftaly et al. employed a THz time-domain trajectory containing secondary peaks to correct or reduce spurious oscillations in the THz spectrum caused by secondary reflection echoes in the samples [8]. However, this method can only correct secondary echoes but not the multiple echoes beyond secondary reflections. Dayou Liu et al. proposed a time-domain optimization method based on the transmission THz time-domain spectroscopy system to correct Fabry-Perot (FP) effects caused by multiple reflections in samples [9]. Moreover, Yang et al. reduced the multiple echoes by adjusting the sample detection transmission model in the transmission THz detection system to obtain theoretical data without multiple echoes [10]. However, THz cannot penetrate the substrates of bonding structures, so the THz detection signals of the samples cannot be obtained through the transmission THz system. L. Wang et al. corrected the multiple echoes using the “signal-adjacent average” method [11]. Although the method is simple to operate, spectral details are lost, which affects the THz detection results. The deconvolution algorithm can only reduce the effects of echoes in the THz time-domain spectroscopy system but cannot correct multiple echoes [12]. P. Yeh et al. assumed a semi-infinite substrate in sample detection. The analytic expressions were constructed in this case. It was found that the essential variations in geometry also led to unpredictable errors in the spectrum [13].

Another reason for generating multiple echoes lies in the multiple reflections of the sample interface reflections in the elements of the THz time-domain spectroscopy system during the THz NDT. Sun et al. adopted the wavelet transform technique to correct the multiple echoes generated from the reflections of THz waves inside the ZnTe crystal in the THz time-domain spectroscopy system [14]. A transmission THz time-domain spectroscopy system was adopted in this study, which did not apply to the NDT for bonding structures. In this paper, multiple echoes recurred at certain intervals and moved within the time window with the changing sample thickness.

Taking the polymethacrylimide (PMI) foam five-layer adhesive structure as an example, the improved wavelet multi-scale analysis method is used to remove multiple echoes according to their frequency characteristics. Compared with the traditional wavelet multi-scale analysis algorithm, it keeps more details of the detection waveform, suppresses the waveform distortion caused by multiple echoes, and corrects errors in the selection of feature peaks and valleys. It solves the problems of time-of-flight image discontinuity caused by multiple echoes and defect area in defect feature imaging, eliminates the influence of multiple echoes caused by system structure in reflective terahertz nondestructive testing, makes the terahertz nondestructive testing results of bonded structures more objective, and indirectly promotes the development of a terahertz nondestructive testing field.

## 2. Introduction to the Detection System and Samples

The Materials and Methods should be described in sufficient detail to allow others to replicate and build on the published results. Please note that the publication of your manuscript implies that you must make all materials, data, computer code, and protocols associated with the publication available to readers. Please disclose any restrictions on the availability of materials or information at the submission stage. New methods and protocols should be described in detail, while well-established methods can be briefly described and appropriately cited.

**Figure 1:**
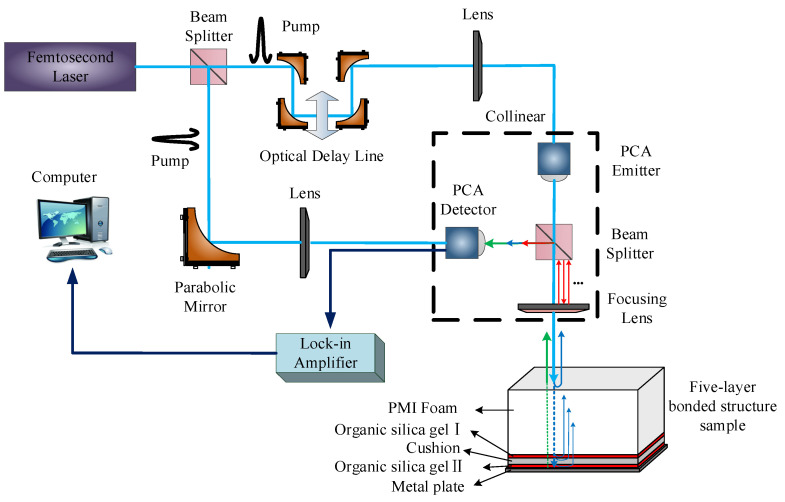
Schematic diagram of the NDT of the THz time-domain spectroscopy system.

The schematic diagram of the NDT of the reflective THz time-domain spectroscopy system is shown in Figure 1, with a time window of 320 ps, a sampling frequency of 0.1 ps, and a spectrum range of 0.1–5 THz. The system mainly consisted of a mode-locking femtosecond laser, an optical delay line, a transmitter, and a receiver. First, the pulse emitted by the femtosecond laser was decomposed by a beam splitter into two beams. One with weak transmission was taken as the probe light and integrated with the THz pulse to pass through the THz detection element collinearly. The other beam with the strong transmission served as the pump light, modulated by the 1.11 kHz chopper and incident on the photoconductive antenna to generate a THz pulse. This pulse was incident on the detection sample after passing through the beam splitter and then the focusing lens. Next, the THz wave, which contained the internal information of the detection sample, passed through the focusing lens and beam splitter to enter the acquisition unit. Finally, the THz detection data of the sample were obtained.

**Figure 2:**
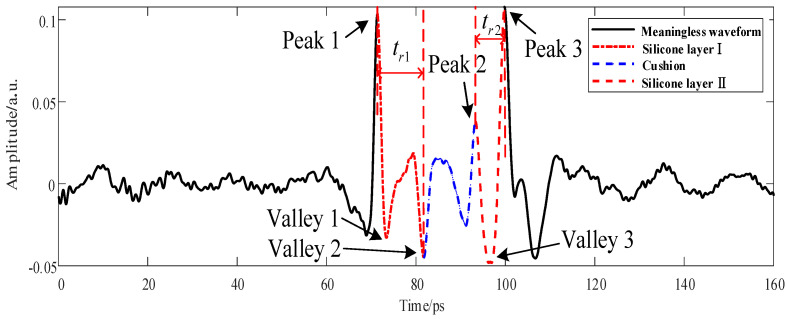
Area division diagram of the adhesive-layer THz waveform of the five-layer bonded structure.

The THz wave propagated in the five-layer bonded structure of PMI foam (Figure 1) to PMI foam, adhesive layer I, cushion, adhesive layer II, and metal plate successively, experiencing reflection or transmission at the interfaces of different media [15]. After the reflection and transmission of the THz wave on the PMI foam surface, the reflected signal was fed back into the THz time-domain waveform signal in the order of propagation time. The transmitted signal continued propagating downward. It underwent total reflection on the metal plate, which was fed back to time-of-flight differences of the THz time-domain waveform based on the sequence of transmission media [16]. As shown in Figure 2, Peak 1, Valley 2, Peak 2, and Peak 3 are the interface echoes between the PMI foam and adhesive layer I, between adhesive layer I and the cushion, between the cushion and adhesive layer II, and between adhesive layer II and the metal plate, respectively. Moreover, the waveform between Peak 1 and Peak 3 is the adhesive-layer waveform of the five-layer bonded structure of PMI foam [6]. The time unit is ps throughout this paper. In addition, only the waveform range of 0–160 ps, which included the adhesive-layer waveform within the time window, was intercepted in the sample detection waveform.

## 3. Problem Analysis

**Figure 3:**
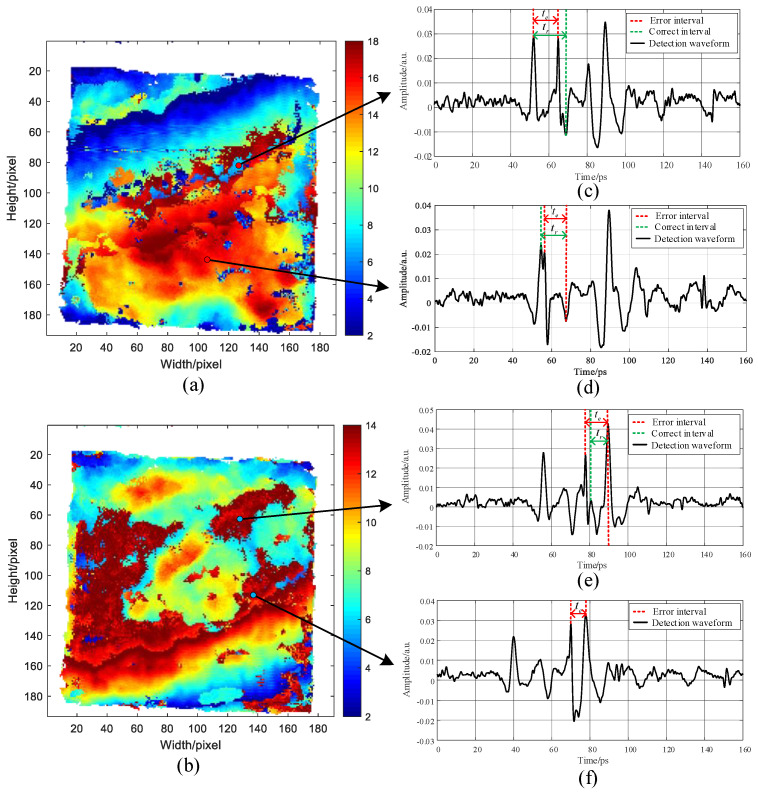
Schematic diagram of time-of-flight results of (**a**) adhesive layer I, (**b**) adhesive layer II, (**c**,**d**) interference waveform of adhesive layer I, and (**e**,**f**) interference waveform of adhesive layer II.

Time-of-flight imaging was performed based on the extraction of the abscissa distance between Valley 2 and Peak 1, $t_{r1}$, and the abscissa distance between Peak 3 and Peak 2, $t_{r2}$, in the THz detection waveform x(t) [17,18], with the results given in Figure 3.

The areas with abnormal chromaticity changes in Figure 3a,b are concentrated in the image’s upper and lower halves, respectively. The leaping changes in image chromaticity were attributed to the influences of the interference signals mixed with the detection signals on the time-of-flight calculation of adhesive layers I and II. In Figure 3c–f, $t_{r}$ denotes the time-of-flight span of the correct characteristic peaks and valleys regarding adhesive layers of the detection waveform, and $t_{e}$ is the time-of-flight span of the wrong characteristic peaks and valleys of the adhesive layers due to the mixing of interference signals. Given the interference signals that led to abnormal changes in image chromaticity, $t_{r}$ was replaced by $t_{e}$, becoming the time-of-flight of the detection data.

Figure 3 shows that the interference signals overlaid different areas of the adhesive-layer waveform, thus excluding echo interference in the sample. The following three assumptions were made according to the propagation paths of the THz reflection echoes in the system: multiple reflections occurred (1) in the focusing lens, (2) in the beam splitter, and (3) between the beam splitter and the focusing lens.

**Figure 4:**
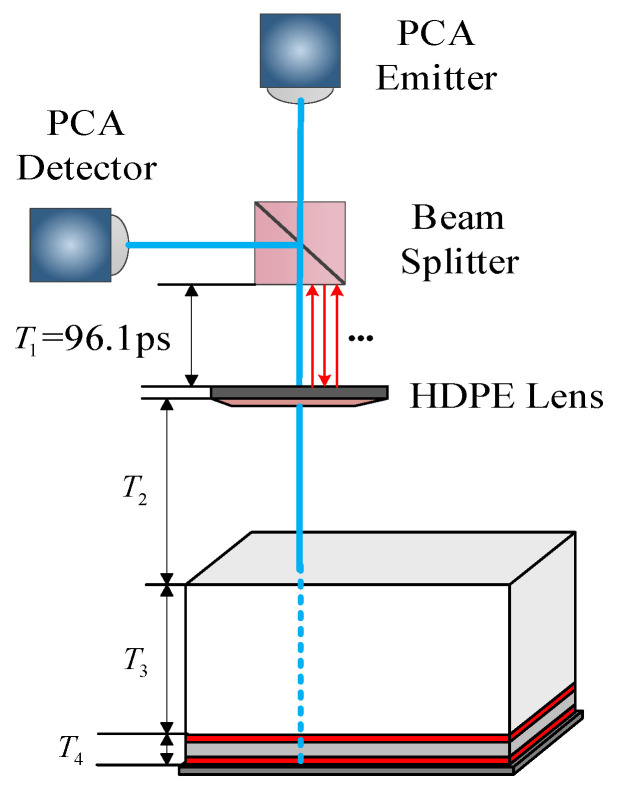
Time-of-flight schematic diagram of THz NDT in the five-layer bonded structure of PMI foam.

A schematic diagram of the time-of-flight of THz NDT was prepared in the five-layer bonded structure of PMI foam based on the above-mentioned assumptions. The Figure 4 $T_{1}$ represents the flight time of the terahertz wave between the beam splitter and focusing lens; $T_{2}$ is the flight time of the terahertz wave between the focusing lens and the upper surface of the sample; $T_{3}$ is the flight time of the terahertz wave in PMI material; $T_{4}$ is the time of flight of the terahertz wave in the adhesive layer of the heat-resistant structure. The THz wave propagated in the beam splitter and the focusing lens for about 25 ps, according to the optical path difference formula. The distance between the beam splitter and the focusing lens was fixed. Hence, the time-of-flight of the THz wave was 96.1 ps.

**Figure 5:**
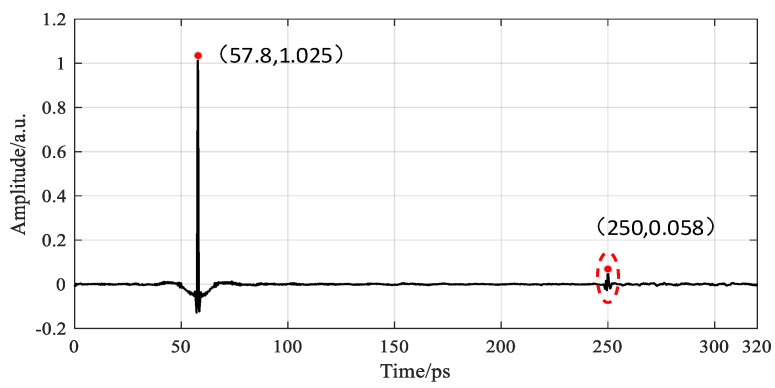
Schematic diagram of the interference signal generated by the metal plate.

To determine the cause of multiple echoes, the above three guesses are theoretically verified: replace the sample with a metal plate and place it on the focus of the focusing lens. Terahertz generates total reflection on the surface of the metal plate. In the time domain signal, it was found that the interference signal appeared 192.2 ps after the main pulse was reflected by the metal plate. As shown in Figure 5, due to the thin thickness of the beam splitter and the focusing lens, the feedback position of the multiple reflection echo of the terahertz wave in the beam splitter and the focusing lens is inconsistent with the position where the multiple echoes appear in the figure [19], but the flight time of the terahertz wave between the focusing lens and the beam splitter is satisfied 2T_1_=192.2 ps. Therefore, it is determined that multiple echoes are generated by the multiple reflections of the terahertz reflection signal between the beam splitter and the focusing lens. If the causes of multiple echoes do not belong to the above three conjectures, the reason for the multiple echos may be due to the defect in PMI foam. The default PMI foam in this paper has no defects, so this situation is not discussed.

The following conditions were assumed to determine the type of the THz reflected signal with multiple reflections between the beam splitter and the focusing lens, including the height of the THz wave from the surface of the detection sample $d_{1}$, the refractive index of air $n_{air}=1$, the average thickness of PMI foam $d_{2}=45$ mm, the thickness of adhesive layer I $d_{3}=0.8$ mm, the thickness of the cushion $d_{4}=4$ mm, and the thickness of adhesive layer II $d_{5}=0.5$ mm. Values $T_{4}=23.92$ ps and $T_{1}>2T_{4}$ were obtained using the optical path difference formula, revealing that the multiple echoes generated by the adhesive layer echo were not mixed in the adhesive-layer waveform of the THz detection waveform. According to the THz propagation path, when the surface echo of the sample satisfied the following conditions, it would mix with the adhesive-layer area of the detection waveform:

(1) $$kT_{1}\in (2T_{3},2(T_{3}+T_{4})),k\in z$$

Therefore, multiple echoes would be mingled in different areas of the adhesive-layer waveform with the change in the sample’s thickness.

## 4. Improved Wavelet Multi-Scale Analysis Algorithm

Considering the unique physical characteristics of the multiple echoes generated in THz NDT, signal processing methods such as wavelet transform and Wiener filtering could not correct the multiple echoes while preserving the detailed information of the signal in the meantime. In the current paper, based on the discrete wavelet db4 [20], a multi-scale analysis was performed to constrain the signal reconstruction range according to the THz propagation time, and it could preserve the detailed information of the signal maximally and correct multiple echoes. The algorithm is described below.

**Figure 6:**
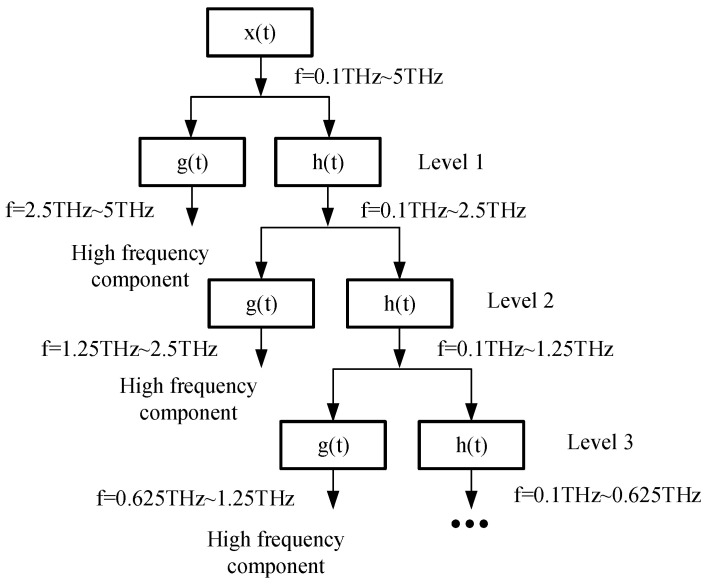
Schematic diagram of multi-scale signal decomposition.

The processed signal x(t) with the frequency range of 0.1 THz~5 THz is multi-scale decomposed through the half band high pass filter g(t) and low-pass filter h(t) to obtain the low-frequency signal $cA_{1}$ and high-frequency signal $cD_{1}$ containing the bonding layer information. Continue to decompose the low-frequency signal $cA_{1}$ to obtain the low-frequency signal $cA_{2}$ and high-frequency signal $cD_{2}$. In this paper, a five-layer multi-scale decomposition is carried out, and the decomposition diagram is shown in Figure 6.

After 2-step sampling of the signal x(t), it can be expressed as the sum of low-frequency components $cA_{j,k}$ and several high-frequency components $cD_{j,k}$ after the last layer of the wavelet decomposition. The formula is as follows:

(2) $$x(t)=cA_{j,k}+\sum_{n}cD_{j,k}$$

where j and k are expansion factors and translation factors, and j,k∈ℤ.

**Figure 7:**
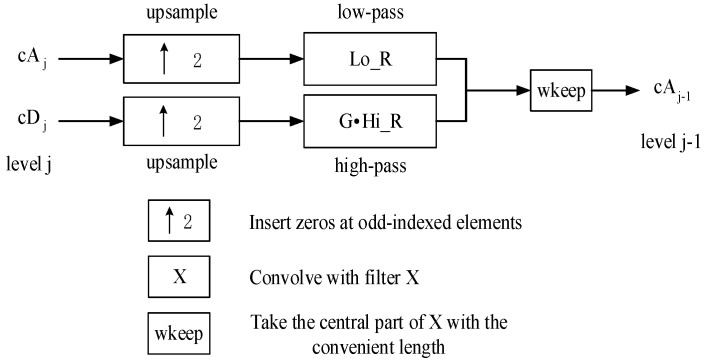
Schematic diagram of signal reconstruction.

The high-frequency components decomposed at each scale contained multiple echoes in the corresponding frequency range and detailed information that could help judge the quality of the bonded structure. Hence, it is not advisable to correct multiple echoes by excluding high-frequency components and only reconstructing low-frequency components [21]. Herein, the time-of-flight result of THz wave propagation was calculated, and the vector of the reconstruction limit range G was added to limit the reconstruction area and retain the details of the waveform, as shown in Figure 7.

The reconstructed signal x(t) was expressed as:

(3) $$X(t)=\sum_{k\in Z}h(t-2k)cA_{j,k}+\sum_{k\in Z}G*g(t-2k)cD_{j,k}$$

where,

(4) $$G=\left\{ \begin{array}{ll} 0, & t\in (t_{1},t_{2})\\ 1, & t\in (0,t_{1})\cup (t_{2},1600) \end{array}\right.$$

$t_{1}$ and $t_{2}$ denote the range of the time domain, where multiple echoes belonged.

## 5. Analysis of Results

### 5.1. Analysis of One-Dimensional Signal Decomposition and Reconstruction

**Figure 8:**
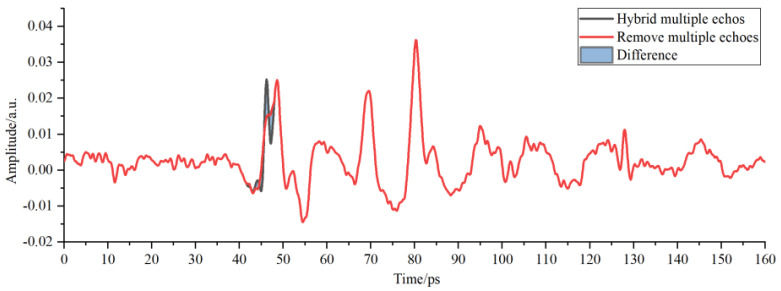
Multi-scale decomposition and reconstruction diagram of the detection waveform of adhesive layer I with hybrid multiple echoes.

**Figure 9:**
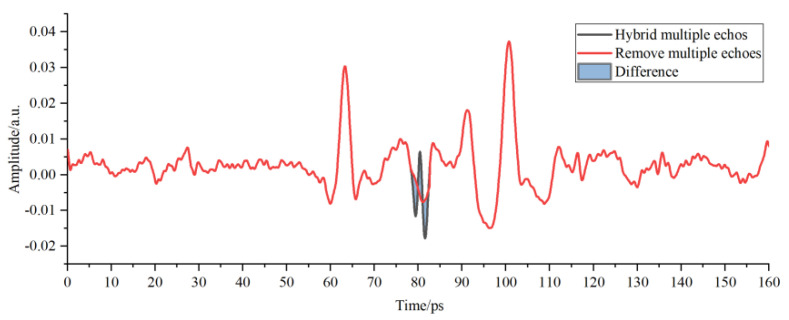
Multi-scale decomposition and reconstruction diagram of the cushion-area waveform with hybrid multiple echoes.

**Figure 10:**
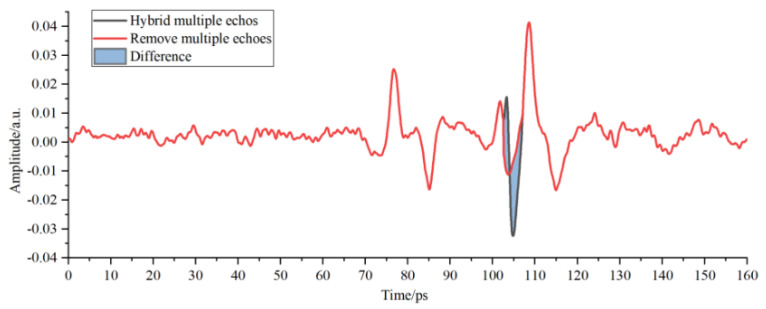
Multi-scale decomposition and reconstruction diagram of the detection waveform of adhesive layer II with hybrid multiple echoes.

In terms of THz reflection echoes at different media interfaces and multiple echoes, their frequency ranges have larger differences as the number of media layers of the bonded structure in PMI foam increases. For this consideration, the improved wavelet multi-scale analysis method was employed to correct the multiple echoes mixed in adhesive layer I, the cushion, and adhesive layer II. The results are shown in Figure 8, Figure 9 and Figure 10.

As shown in Figure 8, the position and amplitude of multiple echoes in the detection waveform were close to those of interface echoes of adhesive layer I in PMI foam, thus influencing the selection of characteristic peaks and valleys of the adhesive layer and causing incorrect judgment of the sample bonding quality. The red waveform represents the results of correcting multiple echoes by the improved multi-scale analysis algorithm in Figure 8. No changes were observed in the position and amplitude of the characteristic peaks and waveform valleys of the adhesive layer within the time window, thereby guaranteeing the analysis objectivity regarding the sample bonding quality of the bonded structure. The THz interface echo between adhesive layer I and the cushion in Figure 9 was mingled with multiple echoes and then drowned in time-domain signals, making it impossible to acquire the amplitude and position within the time window. The THz interface echo between adhesive layer I and the cushion obtained after correcting multiple echoes via the algorithm mentioned above is represented by the red waveform in Figure 9. The peaks of multiple echoes had close positions and amplitudes to those of the THz interface echoes between the cushion and adhesive layer II within the time window. The valleys of multiple echoes increased the valleys of the THz waves in adhesive layer II and caused its deviation to the left within the time window. Then, the multiple echoes mixed in adhesive layer II were corrected through the improved analysis algorithm to obtain the correct THz waveform of adhesive layer II.

As observed in Figure 8, Figure 9 and Figure 10, the hybrid multiple echoes would lead to the rising characteristic peaks of the adhesive layer, the signal submergence of characteristic peaks and valleys, and other results, eventually influencing the judgment of the sample’s bonding quality. Thus, valid information on the detection waveform and multiple echoes were distinguished from the frequency spectrum via the improved wavelet multi-scale analysis algorithm to obtain the original adhesive-layer waveform of the bonded structure.

### 5.2. Analysis of Two-Dimensional Images

**Figure 11:**
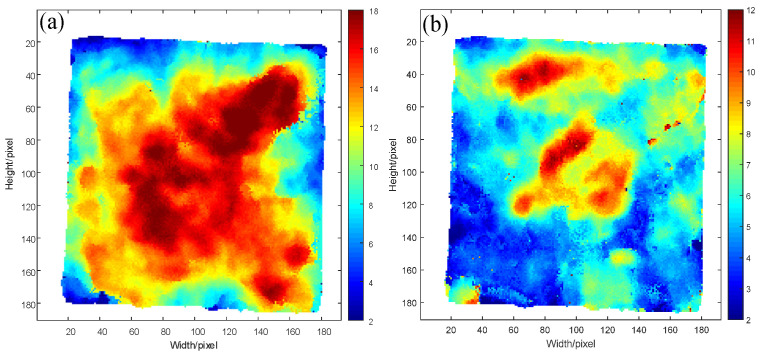
Time-of-flight imaging of THz detection data of (**a**) Time of flight imaging of bonding adhesive layer I after removing multiple echoes, (**b**) Time of flight imaging of bonding adhesive adhesive layer II after removing multiple echoes.

Figure 3a,b show that the introduction of multiple echoes in THz NDT led to significant gradient changes in some image areas and irregular time-of-flight distribution, eventually influencing the NDT results accuracy. The time-of-flight results of adhesive layers I and II after correcting multiple echoes in the detection data by the improved wavelet multi-scale analysis algorithm are depicted in Figure 11a,b.

After the hybrid of multiple echoes in adhesive layer I, the areas with uneven chromaticity distribution were mainly concentrated in the upper half of the image, as shown in Figure 3a. Moreover, many image areas were subject to abrupt chromatic changes represented by abrupt variations in the spacing of characteristic peaks and valleys of adhesive layer I, thereby causing fluctuation in the thickness of this adhesive layer and incorrect time-of-flight results. From the time-of-flight imaging results of adhesive layer I after multiple echoes were corrected in Figure 11a, the gentle gradient changes in the upper part of the image and uniform chromaticity distribution could be observed, thus rectifying the time-of-flight errors due to the hybrid multiple echoes shown in Figure 3a. Figure 3b presents the time-of-flight imaging results of adhesive layer II with hybrid multiple echoes, with abrupt chromaticity changes mainly in the lower half and many dark red areas. The considerable thickness of adhesive layer II influenced the judgment of bonding performance in terms of sample bonding. According to the time-of-flight results of adhesive layer II after correcting multiple echoes in Figure 11b, the red area in the lower half of the image almost disappeared completely, accompanied by uniform chromaticity distribution, more gentle gradient changes, and higher image smoothness.

The mean square error, information entropy, average gradient, and variance of such images were calculated to compare the time-of-flight images before and after completing multiple echoes. The results are listed in Table 1.

According to Table 1, the time-of-flight images of adhesive layers after correcting multiple echoes had smaller values of information entropy, average gradient, and variance than the time-of-flight images of adhesive layers with hybrid multiple echoes. In addition, a smaller chromaticity gap between adjacent pixels in the image, a higher mean square error of the time-of-flight image of adhesive layers compared with the time-of-flight image of adhesive layers with multiple hybrid echoes, and higher smoothness of the overall image were obtained after correction.

**Figure 12:**
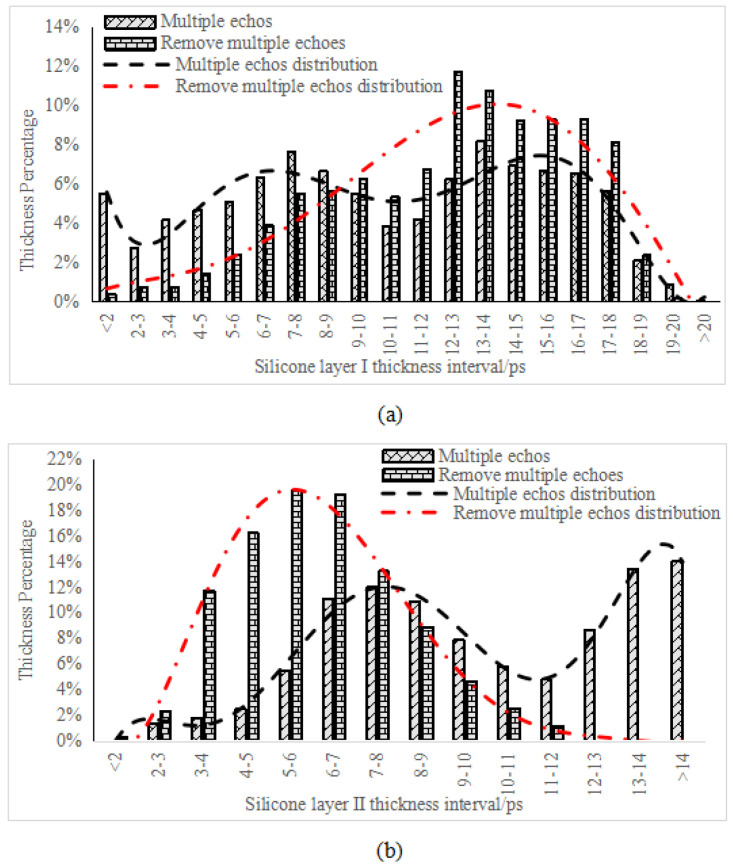
Time-of-flight statistics of (**a**) adhesive layer I and (**b**) adhesive layer II.

For a more intuitive comparison of the time-of-flight images of adhesive layers before and after correcting multiple echoes, the time-of-flight distribution of adhesive layers I and II before and after the correction, as shown in Figure 11, was plotted in Figure 12.

The adhesive layer with hybrid multiple echoes presented an irregular, disordered time-of-flight distribution. In contrast, after correcting multiple echoes, the adhesive layer met the normal distribution, thus following the objective law of the thickness distribution of the bonded sample’s adhesive layers and the criteria of the central limit theorem.

**Figure 13:**
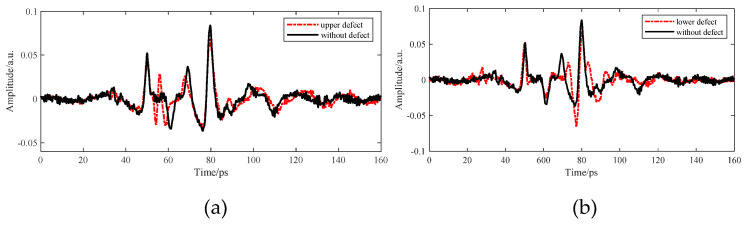
Waveform defect diagram of the five-layer bonded structure. (**a**) Comparison of defect waveforms of adhesive layer I. (**b**) Comparison of defect waveforms of adhesive layer II.

In addition to affecting the adhesive layer thickness, the introduction of multiple echoes in the THz NDT process can also interfere with identifying sample defects. In the case of debonding in adhesive layer I, a peak occurred between the adhesive-layer signals, Peak 1 and Valley 2, attributed to the refractive index differences. In the case of debonding in adhesive layer II, a valley occurred between Peak 2 and Peak 3 [15], as shown in Figure 13.

**Figure 14:**
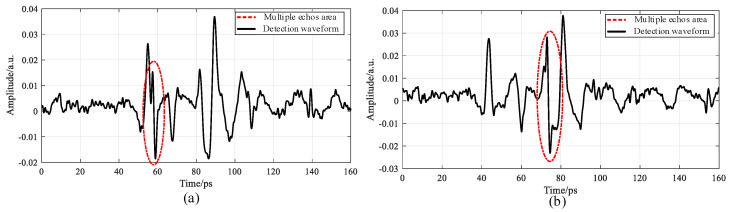
Schematic diagram of characteristic defect identification of adhesive layers with multiple echo interference: (**a**) adhesive layer I and (**b**) adhesive layer II.

When multiple echoes were mingled in the detection data, they could be misjudged as defects during defect identification if the samples had no defects, or they would intensify the debonding degree or interfere with defect identification if the samples had debonding defects, as shown in Figure 14.

**Figure 15:**
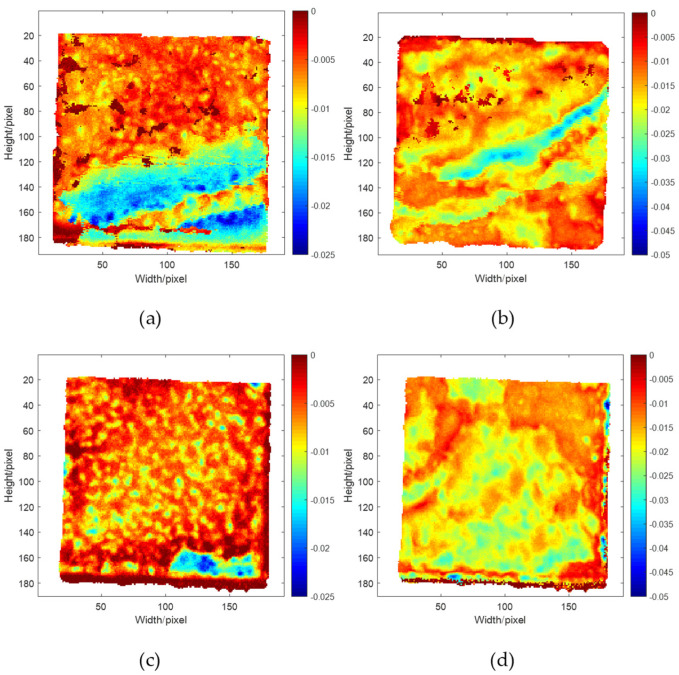
Characteristic defect images of adhesive layers in the PMI foam bonded structure: (**a**) adhesive layer I with hybrid multiple echoes, (**b**) adhesive layer II with hybrid multiple echoes, (**c**) adhesive layer I after correcting multiple echoes, and (**d**) adhesive layer II after correcting multiple echoes.

Characteristic defect imaging of THz detection data was performed [22], and the results are shown in Figure 15.

In the above characteristic defect images, blue and red denote adhesive-layer defects and no defects, respectively. The characteristic debonding defects of adhesive layer I with hybrid multiple echoes were mainly concentrated in the lower half of the image, with a defect area of 21.7%. After correcting multiple echoes, the characteristic defect area was mainly concentrated in the lower right corner, with a defect area of 4.9% and a significant difference in the shape of the debonding area and the area with hybrid multiple echoes. Moreover, the characteristic defects of adhesive layer II with hybrid multiple echoes were mainly distributed in stripes from right to left, with a defect area of 7.3%. In comparison, after correcting multiple echoes, the defects were distributed as star points on the image edge, with a defect area of 2.4%, showing more significant differences in the defect area and distribution area. These findings could be explained by the fact that the test sample thickness satisfied Formula 1. The hybrid multiple echoes in the bonded structure waveform replaced the characteristic defect waveform to interfere with identifying the original defect waveform. Hence, the normal waveform was wrongly identified as the defect waveform in characteristic defect imaging, which expanded the defect area and lowered the accuracy of the THz NDT results.

## 6. Conclusions

This study used the improved wavelet multi-scale analysis method to obtain the correct THz NDT results by limiting the signal reconstruction range, preserving the waveform details, and correcting the distorted detection signal. Then, the mean square error, information entropy, average gradient, and variance between the time-of-flight images of adhesive layers were compared before and after correcting multiple echoes. The results revealed that, compared with hybrid multiple echoes images, the time-of-flight images of adhesive layers after the correction showed higher smoothness and transformed from disordered distribution to normal distribution, thereby satisfying the criteria of the central limit theorem. Hence, correcting the identification errors in characteristic defects caused by multiple echoes guarantees the accuracy of detection results.

## Figures and Tables

**Table 1:** Comparison of time-of-flight images of adhesive layers.

Adhesive Layer Area	Waveform Type	MSE	Entropy	Average Gradient	Variance
Silicone adhesive layer I	Hybrid multiple echoes	21.1387	4.1300	1.7611	22.8844
	Remove multiple echoes		1.6550	0.5273	10.7317
Silicone adhesive layer II	Hybrid multiple echoes	42.3495	3.4221	1.3256	9.8740
	Remove multiple echoes		1.6460	0.4540	3.6729

## Data Availability

The data presented in this study are available on request from the corresponding author.

## References

[1] Wang L., Wang Y., Zhao Z. (2013). The removal of echo signals in terahertz time-domain spectroscopy. J. Infr. Millim. Waves.

[2] Wang Y., Chen L., Chen T., Xu D., Shi J., Ren Y., Li C., Zhang C., Liu H., Wu L. (2018). Interference elimination in terahertz imaging based on inverse image processing. J. Phys. D Appl. Phys..

[3] Pan Z., Wen Y., Zheng X., Cui Y. (2018). Research on bonding defect detection method of aerospace composites based on terahertz image. J. Metrol..

[4] Fukuchi T., Fuse N., Mizuno M., Fukunaga K. (2015). Nondestructive testing using terahertz waves. IEEJ Trans. Power Energy.

[5] Cheng L., Wang L., Mei H., Guan Z., Zhang F. (2016). Research of nondestructive methods to test defects hidden within composite insulators based on THz time-domain spectroscopy technology. IEEE Trans. Dielectr. Electr. Insul..

[6] Zhang D.-D., Ren J.-J., Gu J., Li L.-J., Zhang J.-Y., Xiong W.-H., Zhong Y.-F., Zhou T.-Y. (2020). Nondestructive testing of bonding defects in multilayered ceramic matrix composites using THz time domain spectroscopy and imaging. Compos. Struct..

[7] Hirsch O., Alexander P., Gladden L.F. (2008). Techniques for cancellation of interfering multiple reflections in terahertz time-domain measurements. Microelectron. J..

[8] Naftaly M., Miles R. (2007). A method for removing etalon oscillations from THz time-domain spectra. Opt. Commun..

[9] Liu D., Lu T., Qi F. (2020). A reliable method for removing Fabry-Pérot effect in material characterization with terahertz time-domain spectroscopy. IEEE Trans. Terahertz Sci. Technol..

[10] Yang Y., Dal Forno S., Battiato M. (2021). Removal of Spectral Distortion Due to Echo for Ultrashort THz Pulses Propagating Through Multilayer Structures with Thick Substrate. J. Infrared Millim. Terahertz Waves.

[11] Wang L., Liu Y., Wang Y., Zhao Z. Novel band-notch monopole ultra-wideband antenna with external load. Proceedings of the 2012 International Conference on Microwave and Millimeter Wave Technology (ICMMT).

[12] Liu H., Zhang Z., Zhang C. Enhancement of Sensitivity for Bioanalysis by Liquid Chromatography-Electrospray Mass Spectrometry with Trifluoroacetic Acid in Mobile Phase Using a Suppressor. Proceedings of the 2017 International Conference on Optical Instruments and Technology: IRMMW-THz Technologies and Their Applications.

[13] Yeh P., Hendry M. (1990). Optical waves in layered media. Phys. Today.

[14] Sun Q., Deng Y., Cao S., Yu J., Liu F., Wang C., Xing Q. (2010). Joint time-frequency analysis for removing the spectral interference of terahertz. Spectrosc. Spectr. Anal..

[15] Zhang J., Ren J., Li L., Gu J., Zhang D. (2020). THz imaging technique for nondestructive analysis of debonding defects in ceramic matrix composites based on multiple echoes and feature fusion. Opt. Express.

[16] Wang J., Zhang J., Chang T., Liu L., Cui H.-L. (2019). Terahertz nondestructive imaging for foreign object detection in glass fibre-reinforced polymer composite panels. Infrared Phys. Technol..

[17] Charbon E. Introduction to time-of-flight imaging. Proceedings of the SENSORS, 2014 IEEE.

[18] Giacomantone J., Violini M.L., Lorenti L. (2017). Background Subtraction for Time of Flight Imaging. J. Comput. Sci. Technol..

[19] Jin K.H., Kim Y., Yee D.S., Lee O.K., Ye J.C. (2009). Compressed sensing pulse-echo mode terahertz reflectance tomography. Opt. Lett..

[20] Wang L., Rowan-Robinson M., Yamamura I., Shibai H., Savage R., Oliver S., Thomson M., Rahman N., Clements D., Figueredo E. (2008). Timeline analysis and wavelet multiscale analysis of the AKARI All-Sky Survey at 90 μm. Mon. Not. R. Astron. Soc..

[21] Khani M.E., Arbab M.H. (2022). Translation-Invariant Zero-Phase Wavelet Methods for Feature Extraction in Terahertz Time-Domain Spectroscopy. Sensors.

[22] Zhang J., Ren J., Li L., Gu J., Zhang D., Zhou T. (2021). Defect Identification of Layered Adhesive Structures based on Dynamic Time Warping and Simulation Analysis. Infrared Phys. Technol..

